# Expression change in *Angiopoietin-1* underlies change in relative brain size in fish

**DOI:** 10.1098/rspb.2015.0872

**Published:** 2015-07-07

**Authors:** Yu-Chia Chen, Peter W. Harrison, Alexander Kotrschal, Niclas Kolm, Judith E. Mank, Pertti Panula

**Affiliations:** 1 Neuroscience Center and Institute of Biomedicine, Anatomy, University of Helsinki, Haartmaninkatu 8, Helsinki 00290, Finland; 2 Department of Genetics, Evolution and Environment, University College London, Gower Street, London WC1E 6BT, UK; 3 Department of Ecology and Genetics/Animal Ecology, Uppsala University, Norbyvägen 18D, Uppsala 75236, Sweden; 4 Department of Zoology/Ethology, Stockholm University, Svante Arrhenius väg 18B, Stockholm 10691, Sweden

**Keywords:** brain size, artificial selection, neuro-transcriptome, gene expression, knock down

## Abstract

Brain size varies substantially across the animal kingdom and is often associated with cognitive ability; however, the genetic architecture underpinning natural variation in these key traits is virtually unknown. In order to identify the genetic architecture and loci underlying variation in brain size, we analysed both coding sequence and expression for all the loci expressed in the telencephalon in replicate populations of guppies (*Poecilia reticulata*) artificially selected for large and small relative brain size. A single gene, Angiopoietin-1 (*Ang-1*), a regulator of angiogenesis and suspected driver of neural development, was differentially expressed between large- and small-brain populations. Zebra fish (*Danio rerio*) morphants showed that mild knock down of *Ang-1* produces a small-brained phenotype that could be rescued with *Ang-1* mRNA. Translation inhibition of *Ang-1* resulted in smaller brains in larvae and increased expression of *Notch-1*, which regulates differentiation of neural stem cells. *In situ* analysis of newborn large- and small-brained guppies revealed matching expression patterns of *Ang-1* and *Notch-1* to those observed in zebrafish larvae. Taken together, our results suggest that the genetic architecture affecting brain size in our population may be surprisingly simple, and *Ang-1* may be a potentially important locus in the evolution of vertebrate brain size and cognitive ability.

## 1. Introduction

Vertebrate brain size is remarkably variable at all taxonomic levels. This variation has been proposed to be generated through the balance between positive selection for cognitive ability and the energetic costs of developing and maintaining a larger brain [1,2]. Comparative studies across a range of taxa [3–6] support the link between brain size and cognitive performance, and the human lineage is distinguished in large part by a dramatic increase in relative brain size and cognitive ability [7].

Despite the widespread interest in brain size and cognition, identifying the targets and mechanisms of selection underlying the evolution of vertebrate brain size and function has proved difficult, and little is known about the genetic basis of variation in relative brain size and cognitive ability [8]. In order to identify the causative genetic agents underlying adaptive changes in relative brain size, we used laboratory populations of guppies, *Poecilia reticulata*, that have been subject to artificial selection for either increased or decreased relative brain size [9]. These replicated selection lines showed a 9% difference in relative brain size after two generations of selection, and this difference in brain size was associated with differences in cognitive abilities [9,10].

The rapid convergent response in brain size and cognitive ability observed in each replicate population across just a few generations indicates that standing genetic variation, rather than de novo mutation, was the target of artificial selection in these selection lines. As such, we do not expect as strong a signature of selective sweeps as might be expected with de novo mutations in the loci underlying the phenotypic change. Furthermore, the rapid response from a small starting population suggests a relatively simple underlying genetic architecture, as the relatively small population size makes it unlikely that many variants of relatively small effect would be present in the correct combinations in all replicate lines. Brain-expressed genes show slow rates of functional evolution in general [11,12], and recent evidence suggests that rapid evolutionary changes in the vertebrate brain are the product of expression differences [13,14]. Hence, the response to artificial selection for brain size in the experimental populations is likely the result of shifts in average expression of causative genes.

To investigate the genetic architecture behind guppy brain size variation, we first used full genome expression analysis of adults of the large- and small-brained guppy selection lines. Then, in order to establish functional confirmation of our gene expression results, we knocked down expression of candidate genes in zebrafish (*Danio rerio*) larvae and quantified effects on relative brain size. We also compared our results from zebrafish larvae with *in situ* hybridization analysis of candidate genes in newborn guppies with different brain size.

## 2. Material and methods

### (a) Sample collection and preparation

Brain samples were collected from replicate selected populations and the pre-selected population from sexually mature individuals [9]. After three generations of brain weight selection, body size did not differ between populations, but absolute brain weight showed significant difference; the large-brained males (LBm) showed 3.9% heavier brains than pre-selected males (PSm) of the base population, whereas the small-brained males (SBm) showed 8.1% lighter brains than the Psm. Large-brained females (LBf) exhibited on average 4.0% heavier brains than pre-selected females (PSf), and small-brained females (SBf) showed 2.8% lighter brains than the PSf.

In all cases, the telencephalon was dissected and preserved in RNAlater prior to RNA preparation. In order to obtain sufficient mRNA for RNA-Seq analysis, we constructed non-overlapping same-sex pools of telencephalons, each comprised of three to four individuals. The pre-selected population was assessed with four male and four female pools. The selection experiments were run in triplicate, resulting in three lines independently selected for large brains and three selected for small brains. We constructed one pool for each sex for each selection line in order to differentiate any gene expression differences that were the product of genetic drift or founder effects within single selected populations from convergent changes underlying brain size across replicates. In total, we had 12 selected pools (one male and one female from each of the three large-brain replicate lines and three small-brain replicate lines) and eight (four male and four female) pre-selected pools.

Following RNA extraction (Qiagen RNAEasy lipid tissue kits) using standard manufacturer protocols, RNA samples were prepared and barcoded by the Wellcome Trust Centre for Human Genetics, University of Oxford, using standard protocols. All samples were sequenced on an Illumina HiSeq 2000 as paired-end 100 bp reads.

### (b) Transcriptome assembly and analysis

We assessed the quality of the generated reads using FastQC (http://www.bioinformatics.bbsrc.ac.uk/projects/fastqc), and using Trimmomatic [15] conducted quality filtering and exclusion of read pairs with residual adaptor sequences. Reads were trimmed if the leading or trailing bases had a Phred score of less than 4, and reads were also trimmed if a sliding window average Phred score over four bases was less than 15. Post filtering, reads where either pair was less than 36 bases in length were removed from subsequent analyses, resulting in 16.6 million mappable paired-end reads on average per pool.

All of the samples were combined into a single de novo transcriptome assembly in order to enable orthology determination and comparisons of the generated contigs across the samples. The de novo transcriptome was constructed using Trinity [16] producing 466 694 contigs. Matches to ribosomal RNA sequences were removed prior to mapping to prevent expression bias. To identify which samples expressed each of the contigs and to obtain expression levels, we separately mapped back the filtered reads from each pool to the Trinity contigs using RSEM v. 1.2.4 [17]. In order to remove lowly expressed and erroneous contigs from the de novo assembly, a minimum expression filter of 2 fragments per kilobase per million (FPKM) in at least half of the pools for each sex of each treatment was applied, as has been similarly effectively performed in previous studies [18,19], resulting in 19 698 significantly expressed contigs that were used for further analysis. Of these, 14 227 mapped to the *Xiphophorus* genome [20] (Xipmac 4.4.2 assembly, Ensembl release 72 [21]) using a threshold of *E*^−10^.

To account for differences in the mass composition of the RNA-Seq samples, we conducted trimmed mean of *M*-values (tmm) normalization of expression values using EdgeR [22]. From the 19 698 significantly expressed contigs, differential expression between small- and large-brained population pools was calculated using a twofold expression threshold (log_2_-fold change more than 1 or less than −1) as well as the empirical Bayes estimation and exact tests based on the negative binomial distribution in EdgeR [22], with a *P*_adj_ value less than 0.05, having corrected for multiple testing. The relationship between the samples was assessed with hierarchical clustering implemented in the R package ‘pvclust’ [23], using complete Euclidean distance with 1000 bootstrap replicates and an expression heatmap employing the R package ‘pheatmap’. Coding sequence variant calling was conducted using SAMtools (v. 0.1.19 [24]).

To confirm our de novo assembly results, we also mapped our expression data to the nearest available reference genome, the platyfish, *Xiphophorus maculatus* (Xipmac v. 4.4.2 [20]), obtained from Ensembl release 72 [21]. Mapping was conducted using Tophat 2 (v. 2.0.10 [25]), which leverages the short read aligner Bowtie 2 (v. 2.1.0 [26]). Raw read counts were extracted using HTSEQ-count [27]. Differential expression between small- and large-brained populations was calculated using both a twofold difference in expression (log_2_-fold change more than 1 or less than −1) and a *P*_adj_ value less than 0.05 correcting for multiple testing [22].

### (c) Zebrafish morpholino experiments

Zebrafish were obtained from the breeding line maintained in the Panula laboratory for more than a decade [28]. Fish were raised at 28°C and staged in hours post-fertilization or days post-fertilization (dpf) as described previously [29]. The α1-T-GFP transgenic fish line was a kind gift from Dr Daniel Goldman [30].

The sequence of *Ang-1* antisense morpholino oligonucleotide (MO) (*Ang-1* MO, 5′-GATAGTGCTGTCTTAATATACCTGG-3′; Gene Tools LLC, Philomath, OR, USA) targeting the splicing-donor sites of exon 2 and intron 2 was designed according to Lamont *et al.* [31]. The working concentration was determined by injecting serially diluted MO. At 9 ng MO dose, morphants exhibited small head size and severe cardiac oedema. Therefore, the 6 ng of injection dose, which caused mild gross phenotype that could be rescued by *Ang-1* mRNA, was mainly used in this study. A standard control MO (ctrl MO, 5′-CCTCTTACCTCAGTTACAATTTATA-3′) purchased directly from Gene-Tools was injected 8 ng per embryo. The efficacy of the splice-blocking *Ang-1* MO was analysed by RT-PCR using 3-dpf cDNA as templates with primers (*Ang-1* forward 5′-CAGATTGTGGAGGAGTTGAG-3′ and reverse 5′-AGGTCAGATTTCTCCGTCCG-3′ (*15*); β-actin forward 5′-GGTTTTGCTGGAGATGATGCC-3′ and reverse 5′-CACGGACAATTTCTCTTTCGG-3′). The full-length *Ang-1* cDNA construct was prepared by RT-PCR (primers: forward 5′-AGATCT AAGCTT CCCAGCATCTGCACTCAATCT-3′ and reverse 5′-GATATC CTCGAG GGACTATGAGAAGTCGGCTGG-3′) using and Phusion High-Fidelity PCR Master mix (Finnzymes, Espoo, Finland). The PCR amplicons verified by sequencing with no mutations were cloned into the pMC expression vector kindly given by Dr Thomas Czerny [32]. Capped sense full-length transcripts were generated by the mMESSAGE mMACHINE kit (Ambion, Austin, TX, USA) using T7 RNA polymerase. For the mRNA rescue experiment, 500 pg of angpt1 mRNA with *Ang-1* MO was coinjected into embryos at the one-cell stage (electronic supplementary material, figure S1*a*,*b*).

### (d) Whole-mount *in situ* hybridization

Whole-mount *in situ* hybridization was performed on 4% paraformaldehyde (PFA) fixed 2-dpf zebrafish embryos and dissected 5-dpf brains and followed Thisses' protocol [33]. Antisense digoxigenin (DIG)-labelled RNA probes were generated using the DIG RNA-labelling kit (Roche Diagnostics, Germany), following the manufacturer's instructions. *Notch-1a*, *pax2a* and *pax6a* plasmids were kindly given by Dr Michael Brand. The prehybridization and hybridization were conducted at 65°C for all riboprobes. Guppy *Ang-1* and *Notch-1* sequences were cloned with RT-PCR from two adult male guppy brains. Primer sequences were *Notch-1* forward 5′-GCACAACCAGACTGACCGTA-3′, *Notch-1* reverse 5′-CTATGCTGGGAGGGAGGAGT-3′, *Ang-1* forward 5′-GATGGCTCACCTGCAGCAGA-3′ and *Ang-1* reverse 5′-GCAGCTCCTGATTGGTTGGA-3′. The PCR amplicons were cloned into the pGEM-T Easy vector (Promega, Madison, WI, USA) and verified by sequencing. The *Notch-1* and *Ang-1* antisense riboprobes were synthesized by SP6 RNA polymerase with plasmids linearized by SacII.

Samples from at least three individual large- and small-brain parents (*n* = 8–10 for each group) were examined. Guppy fry were scarified during the day they were born and fixed in 4% PFA in PBS buffer at 4°C overnight. After fixation, the fixed brains were dissected from heads. The *in situ* hybridization was done as previously described [34]. The hybridization was done at 62°C and washed at 65°C. *In situ* hybridization signals were detected with sheep anti-digoxigenin-AP Fab fragments (1 : 10 000; Roche Diagnostics). The colour staining was carried out with chromogen substrates (NBT and BCIP).

### (e) RNA isolation, cDNA synthesis and quantitative real-time PCR

Total RNA was extracted from 30 pooled 3-dpf embryos or 25 pooled 6-dpf heads (RNeasy mini Kit; Qiagen, Valencia, CA, USA). To synthesize cDNA, 2 µg total RNA were reverse-transcribed using SuperScriptTM III reverse transcriptase (Invitrogen, Eugene, OR, USA) according to instructions provided by the manufacturer.

Quantitative real-time PCR (qPCR) was performed in the LightCycler 480 instrument (Roache, Mannheim, Germany) using the Lightcycler^®^480 SYBR GreenI Maxter (Roache). Primers for amplification were designed by Primer-BLAST (NCBI). Three housekeeping genes, β-*actin*, *elf1a* and ribosomal protein L13a (*rpl-13a*), were used as reference controls. β-*actin* primers were obtained from the Real-time PCR Primer Databank (http://medgen.ugent.be/rtprimerdb/). All primers sets were confirmed to amplify only a single product of the correct size. Sequences of primers were: β*-actin*, 5′-CGAGCAGGAGATGGGAACC-3′ and 5′-CAACGGAAACGCTCATTGC-3′; *elf-1a*, 5′-CCAACTTCAACGCTCAGGTCA-3′ and 5′-CAAACTTGCAGGCGATGTGA-3′; *rpl-13a*, 5′-AGAGAAAGCGCATGGTTGTCC-3′ and 5′-GCCTGGTACTTCCAGCCAACTT-3′; *Notch-1a*, 5′-AGAGCCGGATTCAGCGGTC-3′ and 5′-TTACAGGGACGTGGAGAACAAG-3′). Cycling parameters were as follows: 95°C for 5 min and 45 cycles of the following, 95°C for 10 s, 60°C for 15 s and 72°C for 20 s. Fluorescence changes were monitored with SYBR Green after every cycle. Dissociation curve analysis was performed (0.1°C s^−1^ increase from 60 to 95°C with continuous fluorescence readings) at the end of cycles to ensure that only single amplicon was obtained. All reactions were performed in duplicates and three biological replicates were done for each group (*n* = 9). Results were evaluated with the LightCycler 480 software. Cycle thresholds (Ct) obtained from each duplicate were averaged and normalized against the Ct values of β-*actin*, *elf-1a* and *rpl-13a*, respectively, as the reference control [35]. As the gene expression changes showed the same trend when normalized to different housekeeping genes (data not shown), the result referred to β-*actin* was shown in this study. Means and standard deviations were calculated. Statistical differences among groups were analysed using a one-way ANOVA, followed by Dunnett's test (GraphPad software Inc., San Diego, CA, USA). Differences were considered statistically significant at *p* < 0.05.

### (f) Immunocytochemistry and imaging

The whole-mount immunostaining was performed on 4-dpf zebrafish larvae with 2% PFA fixation. Antibody incubation was carried out with 4% normal goat serum and 1% DMSO in 0.3% Triton X-100/PBS for 16 h at 4°C with gentle agitation. The primary antibody was chicken anti-green fluorescent protein (1 : 750; A10263, Invitrogen). The following secondary antibodies were Alexa Fluor^®^ 488 goat anti-mouse or anti-rabbit IgG (1 : 1000; Invitrogen).

Bright-field images were taken with a Leica DM IRB inverted microscope with a DFC 480 charge-coupled device camera and *z*-stacks were processed with Leica Application Suite software and Corel DRAW X3 software [34]. Immunofluorescence samples were examined using a Leica TCS SP2 AOBS confocal microscope. For excitation, an Argon laser (488 nm) was used, and emission was detected at 500–550 nm [36]. Stacks of images taken at 0.2–1.2 μm intervals were compiled, and the maximum intensity projection algorithm was used to produce final images with Leica Confocal Software and Imaris imaging software v. 6.0 (Bitplane AG, Zurich, Switzerland).

### (g) Brain size measurement

Brains of larvae are too small to be removed and weighed. We therefore measured optic tectum width from dorsal digital microscopic images (using ImageJ) as an accurate predictor of overall brain size [37,38] (as, in zebrafish, the width of the optic tectum predicts the mass of the brain with 79% accuracy [39]). We used total length (from the tip of the snout to the tip of the tail) as a body size measure for 64 individual zebrafish (control (18), *Ang-1* MO (18) and *Ang-1* rescue RNA (28)). We analysed body size using a one-way ANOVA with total length as dependent variable and treatment groups as factor. We analysed relative brain size using an ANCOVA with brain size as dependent variable, body size as covariate and treatment group as factors, followed by a post hoc LSD test (SPSS 13.0 software package, SPSS Inc.)

## 3. Results

### (a) Identification of *Ang-1* as an important gene underlying variation in brain size

**Figure 1.**
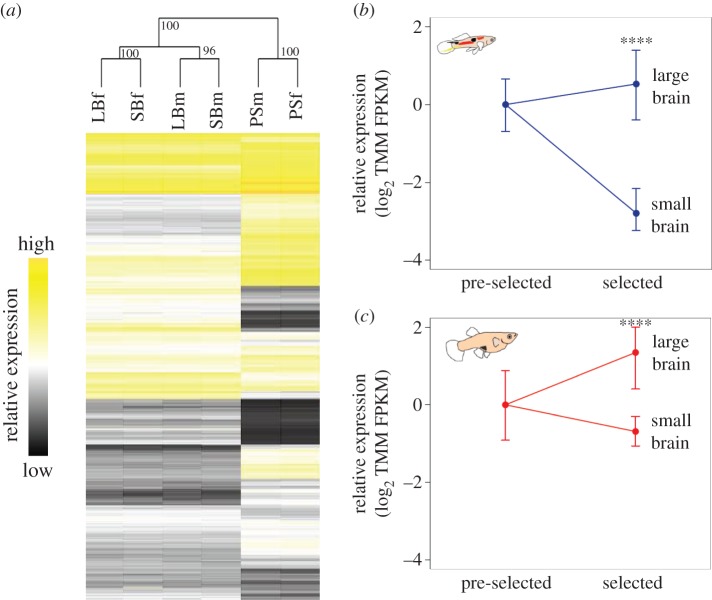
Expression profiles of guppy populations. (*a*) Hierarchical clustering of gene expression across all significantly expressed genes in LBf, LBm, SBf, SBm and PSm, and PSf telencephalons. Clustering is based on Euclidian distance of expression level, with significance provided by 1000 bootstrap replicates shown on each node. Yellow indicates relative higher and black relative lower expression for each transcript. (*b*) Relative expression of *Ang-1* in pre-selected and selected male populations. (*c*) Relative expression of *Ang-1* in pre-selected and selected female populations. In both (*b*,*c*), expression is based on log_2_ relative trimmed mean of *M*-values (TMM) normalized FPKM mapped reads. Tails indicate minimum and maximum expression values for each population. Significance of expression difference between large- and small-brained population pools, assessed with a *t*-test, is indicated in each panel (****P_adj_ < 0.0001).

Within the selected populations, overall transcriptional similarity was stronger for sex than brain size. Hierarchical clustering [23] indicates that the selected populations are more similar to each other in overall expression than they are to the pre-selected population (figure 1*a*). This was further supported by the approximately unbiased *p*-value separating selected from pre-selected populations (based on bootstrapping of expression data, *p* < 0.05). Furthermore, 13 075 transcripts, corresponding to 8639 genes in *Xiphophorus*, showed significant differences in expression between pre-selected and selected populations, regardless of brain size (log_2_-fold change more than 2, *P*_adj_ < 0.05). Environmental complexity has been shown to affect broad patterns of neural gene expression [40], and the differences we observe in overall transcription between the selected and pre-selected populations are likely an effect of holding conditions, with the pre-selected populations kept within large 100 l aquaria at relatively high density. By contrast, each male–female pair of the selected populations was kept in a 3 l aquarium with occasional offspring removed within hours after birth. The transcriptional differences between the pre-selected and selected populations, although not the focus of this study, illustrate the remarkable effects of social environment and intra-sexual social interactions on overall telencephalon transcription.

Importantly, only one gene, *Angiopoietin-1* (*Ang-1*), differed significantly in expression (average log_2_-fold change more than 1 or less than −1, *P*_adj_ < 0.05) between adults in the large- and small-brain replicate populations, showing an average log_2_-fold change of 6.81 between large- and small-brained populations (*P*_adj_ = 4.83 × 10^−18^). Compared to the pre-selected population, our data indicate that *Ang-1* expression increased in all replicate large-brained populations (average log_2_-fold change = 1.81, *P*_adj_ = 3.79 × 10^−2^) and decreased in average expression in all replicate small-brain populations (average log_2_-fold change = −3.75, *P*_adj_ = 4.11 × 10^−9^). To confirm our findings, we also mapped our RNA-Seq data to the nearest available reference genome, the platyfish. As with the de novo analysis, *Ang-1* was also significantly differentially expressed (average log_2_-fold change = 3.1 between large- and small-brained populations, *P*_adj_ = 4.65 × 10^−7^).

We assessed the polymorphism and sequence data from our RNA-Seq data. We found no evidence of fixed differences in *Ang-1* coding sequence between large- and small-brained populations, nor between selected and pre-selected populations. In order to assess the potential for differences in copy number between the selection lines, we tested for excessive haplotypes in our read data and in the corresponding polymorphism data. Although we cannot rule out very recent duplications, there was no indication of differences in copy number based on polymorphism and haplotype data, suggesting that variation in *Ang-1* expression is not owing to copy number variation. The continuous range in expression we observe also suggests that variation is not owing to gene dose effects, which for any one locus would result in more discrete expression categories.

Previous work on these populations recovered sex-specific differences in response to selection for relative brain size and cognitive ability [9], and it is known that brain structure shows sex-specific patterns [41,42], therefore we also analysed adult males and females separately for *Ang-1* expression (figure 1*b*,*c*). In both sexes, *Ang-1* expression was intermediate in the original pre-selected population, and associated with relative brain size in selected populations of guppy. Expression was significantly higher in large-brain populations for both males (log_2_-fold change = 10.70, *P*_adj_ = 8.12 × 10^−21^) and females (log_2_-fold change = 4.42, *P*_adj_ = 6.03 × 10^−11^) compared with small-brain populations. This suggests that changes in relative brain size in the independent replicate populations are the result of convergent selection in expression of *Ang-1*.

### (b) Confirmation of the importance of *Ang-1* expression for brain size development in zebrafish

**Figure 2.**
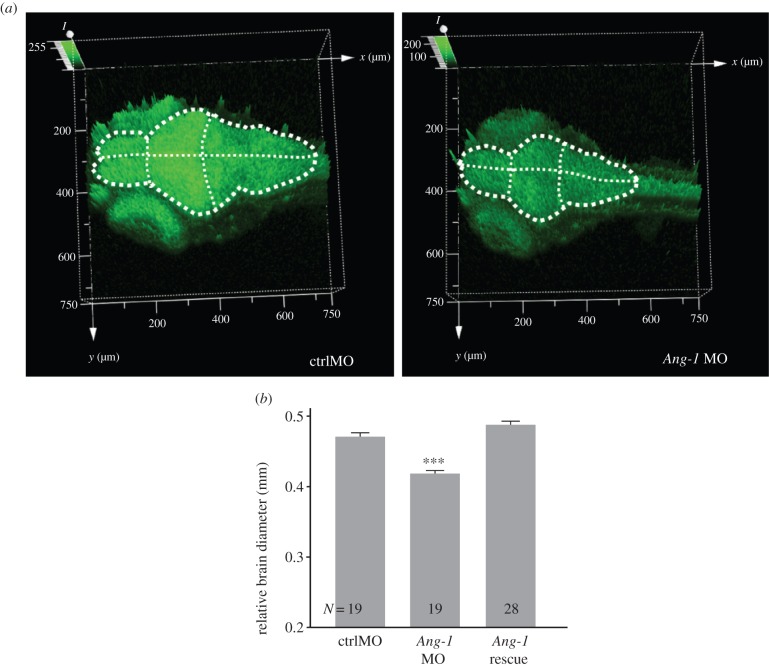
Brain morphology of zebrafish *Ang-1* morphant. (*a*) Knockdown of *Ang-1* expression caused the small-sized brain and the reduction of GFP intensity in the *Ang-1* morphant (*Ang-1* MO) compared with the control group (ctrlMO) in the Tg (alpha-tubulin-GFP) background line at 4 dpf (*n* = 6). Dashed lines illustrate the brain area. (*b*) *Ang-1* MO shows the significantly smaller relative brain diameter (controlled for body size) than the control and rescue group (ANCOVA: group: *F*_2,62_ = 74.55, *p* < 0.001, body length: *F*_1,62_ = 14.47, *p* < 0.001; *post hoc* pairwise group comparisons: ctrlMO versus *Ang-1* MO: *p* < 0.001, ctrlMO versus *Ang-1* rescue: *p* = 0.069, *Ang-1* MO versus *Ang-1* rescue: *p* < 0.001; ***p < 0.001).

**Figure 3.**
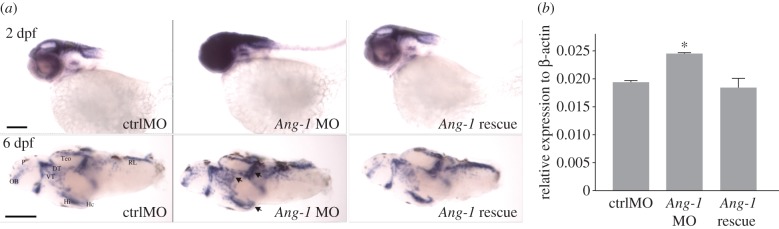
*Notch-1a* mRNA expression. (*a*) *Notch-1a* expression is dramatically increased in 2-dpf *Ang-1* MO morphant brains and 6-dpf brains in the pallium, thalamus, medial and later domains of tectum opticum and intermediate and caudal hypothalamus (*n* = 6–8 each group). The *Ang-1* mRNA normalizes the overexpression of *Notch-1a* in *Ang-1* MO morphants. (*b*) qPCR analysis of *Notch-1a* transcript levels (*p < 0.05, *n* = 9, one-way ANOVA with Dunnett's test). DT, dorsal thalamus; Hc, caudal hypothalamus; Hi, intermediate hypothalamus; OB, olfactory bulb; P, pallium; RL, rhombic lip; TeO, tectum opticum; VT, ventral thalamus. Scale bar, 100 μm.

In addition to known functions in vascular development and angiogenesis [43,44], *Ang-1* has recently been implicated in neuronal growth and development [43,45–47], and therefore presents a compelling candidate gene to explain the variation in brain size and cognitive ability observed in these guppy populations. In order to verify the potential role of *Ang-1* in neural growth and development and to increase the generality of the analysis, we therefore conducted knockdown experiments using translation inhibition with MOs in zebrafish. Although the morphant fish displayed statistically significantly shorter total body length, this was not a substantial difference in size (ANOVA: group: *F*_2,63_ = 51.92, *p* < 0.001; mean ± s.e.; control 4.03 mm ± 0.01 mm; *Ang-1* MO 3.81 mm ± 0.03 mm; *Ang-1* rescue 3.72 mm ± 0.02 mm). Importantly, they also displayed smaller relative brain size relative to controls (figure 2*a*,*b*, and electronic supplementary material, figure S1*c*). Expression of intermediate filament nestin as well as two transcription factors, *Pax2a* and *Pax6a* (pair box proteins 2a and 6a), involved in neurogenesis and brain development [41–43], was not statistically different between the MO and controls (electronic supplementary material, figure S2). However, the cell membrane-tethered transcription factor *Notch-1* showed higher expression in the *Ang-1* morphants than in control MO-injected fish (figure 3*a*,*b*; *F*_2,6_ = 8.01, *p* = 0.02). Our data support previous assessments showing that although *Notch-1* is important during brain development, it is generally lowly expressed in adult tissues [46,48]. *Notch-1* expression in adult guppy telencephalons was relatively low (in the lower 14th percentile of significantly expressed contigs) and did not differ significantly between the large- and small-brained populations (*Notch-1* log_2_ gene expression levels: large-brained pools = 5.93, small-brained pools = 5.52, *P*_adj_ > 0.5). This suggests that changes in *Ang-1* act during development to influence *Notch-1* expression, however *Ang-1* may also act on adult brains independently of the *Notch-1* pathway owing to the indeterminate growth patterns that characterize fish.

### (c) *In situ* analysis of *Ang-1* and *Notch-1* in newborn guppies with large and small brain size

**Figure 4.**
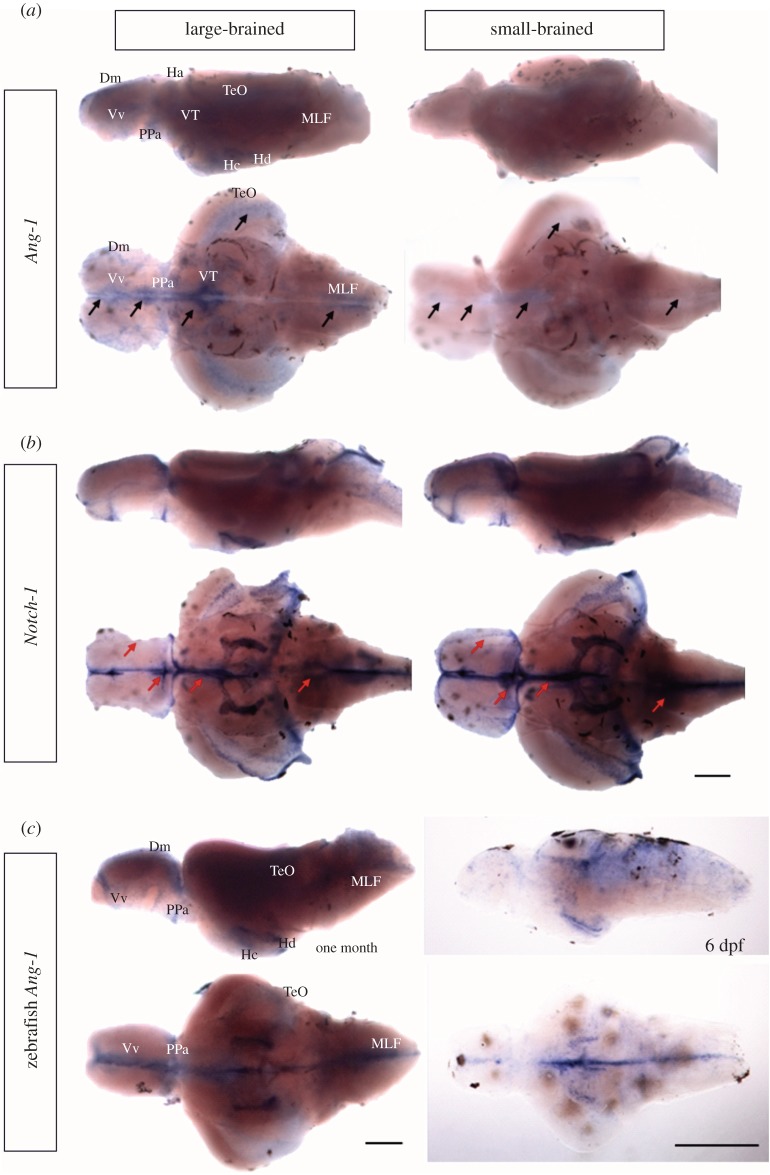
*Ang-1* and *Notch-1* expression in the guppy fry brains. (*a*) Expression of guppy *Ang-1*. (*b*) Expression of guppy *Notch-1*. Both *Ang-1* and *Notch-1* mRNA are present in the medial zone of dorsal telencephalic area (Dm), the ventral nucleus of ventral telencephalic area (Vv), parvocellular preoptic nucleus (PPa), habenula (Ha), ventral thalamus (VT), caudal zone of periventricular hypothalamus (Hc) and dorsal zone of periventricular hypothalamus (Hd), tectum opticum (TeO) and medial longitudinal fascicle (MLF). (*c*) *Ang-1* expression pattern in one-month-old and 6-dpf zebrafish brain. Zebrafish *Ang-1* expression is present in Dm, Vv, PPa, Hc, Hd, TeO and MLF. Black and red arrows, respectively, indicate *Ang-1* and *Notch-1* mRNA difference between the large-brain and the small-brain guppy populations. Scale bars, 200 µm.

Fry from large- and small-brain selection lines differ at birth in relative brain size [9], and we therefore confirmed our zebrafish developmental findings by performing *in situ* hybridizations of *Ang-1* and *Notch-1* on guppy newborn whole brain samples from our selection lines (figure 4). The staining results were consistently reproducible and no signal was present in samples hybridized with sense probes. *Ang-1* transcripts were mainly found in ventricular zones along the rostrocaudal axis of the brain. Signal was also present in the dorsal and ventral telencephalon, the preoptic region, tectum, the periventricular ventral and caudal hypothalamus and medulla. The large-brain guppies showed a higher *Ang-1* expression than small-brain samples. This was particularly evident in the telencephalon. *Notch-1* was found in the ventricular zones of the dorsal, dorsal-medial and ventral telencephalon, the preoptic region, the anterior dorsal and the ventral thalamus, along the periventicular ventral hypothalamus throughout its entire rostrocaudal extent, tectum, cerebellum and rhombencephalon. In comparison with large-brain guppies, samples from small-brain populations showed stronger *Notch-1* mRNA expression in the telencephalon, hypothalamus and medullary areas where *Ang-1* mRNA expression was lower than guppies from large-brain populations. The expression pattern of zebrafish *Ang-1* was examined using *in situ* hybridization on the one-month-old and 6-dpf zebrafish brain. The regions expressing zebrafish *Ang-1* were similar to those for guppy *Ang-1*, mainly in ventricular zones and along the rostrocaudal axis of the brain.

These results show that mRNA distributions of *Ang-1* and *Notch-1* are conserved between zebrafish and guppy brains. Both guppy *Ang-1* and *Notch-1* transcripts were ventricularly located along the rostrocaudal axis of telencephalon, diencephalon and medulla where these areas are identified as important proliferation zones for embryonic and adult neurogenesis in zebrafish [49,50]. Guppies from large-brain populations exhibit higher *Ang-1* expression and lower *Notch-1* expression compared with guppies from small-brain populations, in agreement with the concept that *Ang-1* and *Notch-1* play crucial roles in the regulation of brain size.

## 4. Discussion

Our work suggests that expression changes in *Ang-1* underlie much of the variation in relative brain size in our guppy populations, and our experimental analyses of the effect of *Ang-1* in zebrafish demonstrate its functionality and provide generality in our findings across distantly related teleost species. Although also influenced by environmental effects and developmental noise, the range of expression levels (figure 1*a*–*c*) suggests polygenic variation in *Ang-1* expression, possibly through multiple *cis*-regulatory variants and *trans*-acting factors. *Ang-1* is known to interact with several other genes [51], and although no other genes showed any variation in expression consistent with brain size in our adult animals, they may still play a role early in development and/or in modulating *Ang-1. Ang-1* may also affect brain size via the *Notch-1* signalling pathway during early development, as evidenced by the increase in *Notch-1* mRNA in *Ang-1* morphants and the increase in *Notch-1* expression in the newborn small-brained guppies. *Notch-1* is involved in regulation of neural stem cell differentiation and it may thus be an essential factor in *Ang-1*-mediated regulation of brain size. In zebrafish, the absence of *Psen-1*, a component of the cell membrane gamma-secretase complex that regulates *Notch-1* processing, leads to increased numbers of neurotransmitter-specific neurons (histaminergic neurons) and an alert/anxious behavioural phenotype [52], suggesting that *Notch-1* is an important factor in brain neuron development.

*Ang-1* is not one of the micro-encephaly genes typically studied in relation to human brain size [53–55]. Nevertheless, its potential role in neural proliferation and neural density [45,47,56] makes it a plausible target for selective forces acting on relative brain size and cognitive evolution in natural and human populations [57]. Interestingly, *Ang-1* also promotes angiogenesis [43,44,58]. The observed *Ang-1* expression differences may therefore be caused by the fact that a large brain requires more blood vessels to ensure adequate blood supply than a small brain. Because this may also explain smaller brains in zebrafish *Ang-1* morphants, more targeted experiments will need to determine whether differential *Ang-1* expression leads to a change in brain size via a direct effect on neural growth and development [43,45–47], or indirectly via a change in angiogenesis [43,44]. It will be interesting to see how selection for changes in expression of *Ang-1* in relation to brain size affects its other functions.

Future investigations of expression levels of *Ang-1* across other taxa are needed to test the generality of our findings in vertebrates. Such studies will also offer a novel addition to previous macroevolutionary studies (e.g. [2,4,59,60]) of ecological correlates of variation in brain size across the animal kingdom. The range of *Ang-1* expression and rapid change in average expression over the short-time period indicates that artificial selection acted on a substantial pool of standing genetic variation present in the pre-selected population. This predicts that rapid change in relative brain size and cognitive ability of wild populations is possible when strong selective forces are present. Finally, our results suggest that *Ang-1* may be of potential importance in the evolution of vertebrate brain size and cognitive ability.

## 5. Conclusion

Based on a combination of artificial selection, whole genome transcriptome analysis and functional genetics in two different fish species, we find that the genetic architecture affecting brain size is surprisingly simple and propose that *Ang-1* is a key gene behind evolutionary changes in vertebrate brain size and cognitive ability.

## Supplementary Material

Chen et al brain size gene supplemental material Proc Roy

## Supplementary Material

suppl Figure 1

## Supplementary Material

suppl Figure 2

## Supplementary Material

Data file

## Data accessibility

Raw Illumina Reads: NCBI Sequence Read Archive Project accession no. PRJNA284588. Data for morpholino zebra fish brains and guppy *in situ*: see the electronic supplementary material.
