# Supplementary material for: Expression change in Angiopoietin-1 underlies change in relative brain size in fish
Source: Proc Biol Sci. 2015 Jul 7;282(1810):20150872. doi: 10.1098/rspb.2015.0872 (PMC4590489; doi:10.1098/rspb.2015.0872)
Supplement: Chen et al brain size gene supplemental material Proc Roy [file rspb20150872supp1.docx]

**Expression change in *Angiopoietin-1* underlies change in relative brain size in fish**

Yu-Chia Chen*^a^, Peter W. Harrison*^b^, Alexander Kotrschal*^c,d^, Niclas Kolm†^c,d^, Judith E. Mank†^b^, Pertti Panula†^a^

* equal contribution

† equal contribution

^a^Neuroscience Center and Institute of Biomedicine, Anatomy, University of Helsinki, Haartmaninkatu 8, 00290 Helsinki, Finland.

^b^Department of Genetics, Evolution and Environment, University College London, Gower Street, London, WC1E 6BT, United Kingdom.

^c^Department of Ecology & Genetics/Animal Ecology, Uppsala University, Norbyvägen 18D, SE-75236 Uppsala, Sweden.

^d^Department of Zoology/Ethology, Stockholm University, Svante Arrhenius väg 18B, SE-10691 Stockholm, Sweden.

Corresponding author: [Niclas.kolm@zoologi.su.se](mailto:Niclas.kolm@zoologi.su.se)

0046 8 164050

**Supplementary Figure 1. Morpholino efficacy and dose-dependent gross phenotype.** **Panel A.** Efficacy of splice-blocking morpholino oligonucletides (MO). Transcripts of 3-dpf control and Ang-1 morphants were used to determine the MO knockdown efficiency by RT-PCR. The upper band (black arrow) indicates the regular Ang-1 transcript. The lower band (red arrow) depicts the aberrant splicing variant. PCR products were verified by sequencing. The β-actin was used as an internal control. **Panel B**. Bright-field images of control, *Ang-1* morphants and *Ang-1*rescue larvae at 6 dpf. **Panel C**. Body-length measurement of 6-dpf larvae. **: *P* < 0.01 by one-way ANOVA with Dunnett’s test. Ang-1MOL: 6 ng of MO; Ang-1MOH: 9 ng of MO; Ang-1RNA: Ang-1 mRNA 500 pg; Ang-1MOL-RS: 6 ng of MO with 500 pg mRNA; Ang-1MOH-RS: 9 ng of MO with 500 pg mRNA. Scale bar is 1 mm.

**Supplementary Figure 2. pax2a, pax6a and nestin expression pattern are intact in 2-dpf Ang-1 morphants (n = 6 each group).** Scale bar is 100 μm.
