# Supplementary figures and images for: Expression change in Angiopoietin-1 underlies change in relative brain size in fish
Source: Proc Biol Sci. 2015 Jul 7;282(1810):20150872. doi: 10.1098/rspb.2015.0872 (PMC4590489; doi:10.1098/rspb.2015.0872)

Supplements Figure 1.

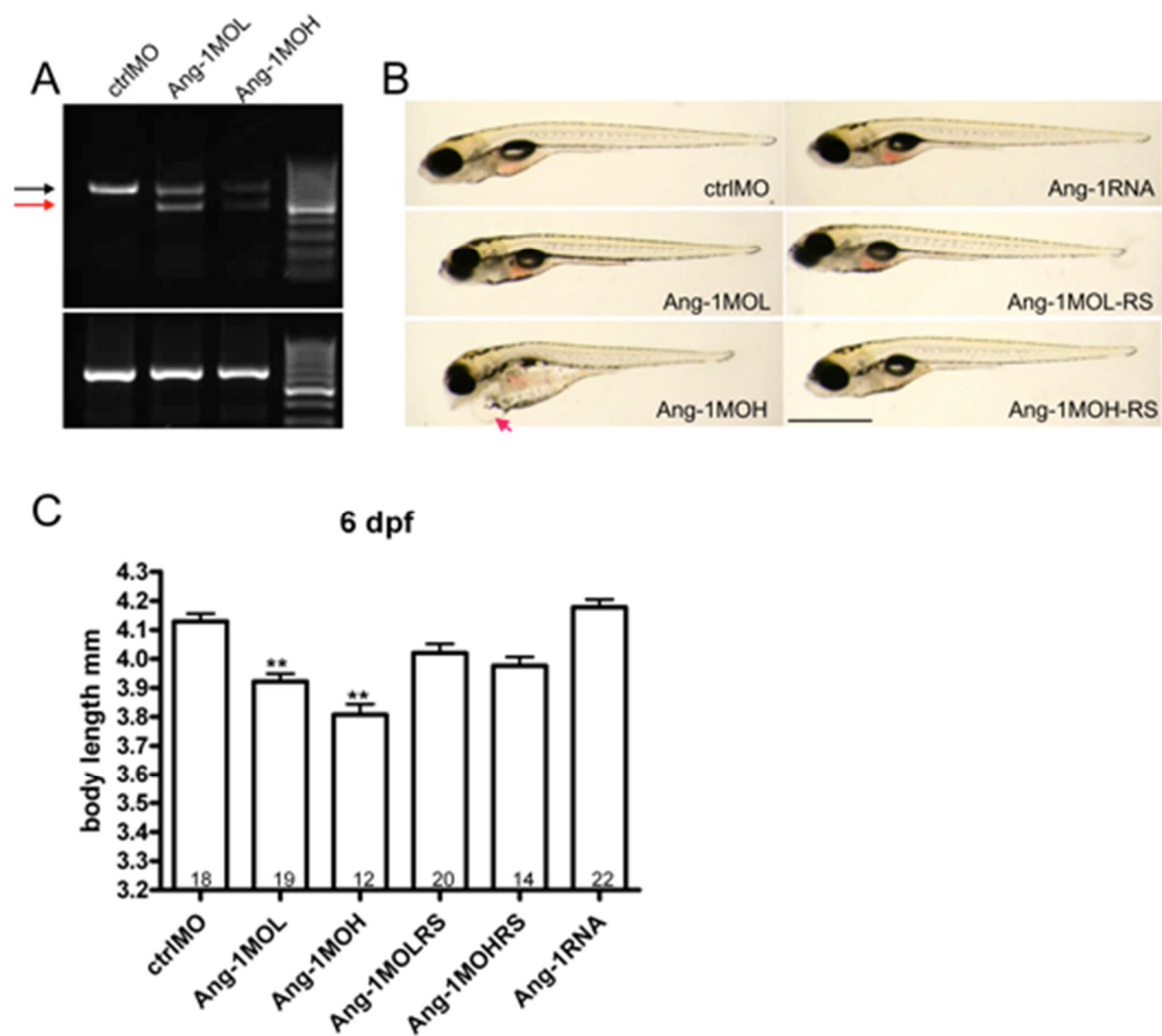

Supplement: suppl Figure 1 [file rspb20150872supp2.pdf]

Supplements Figure 2.

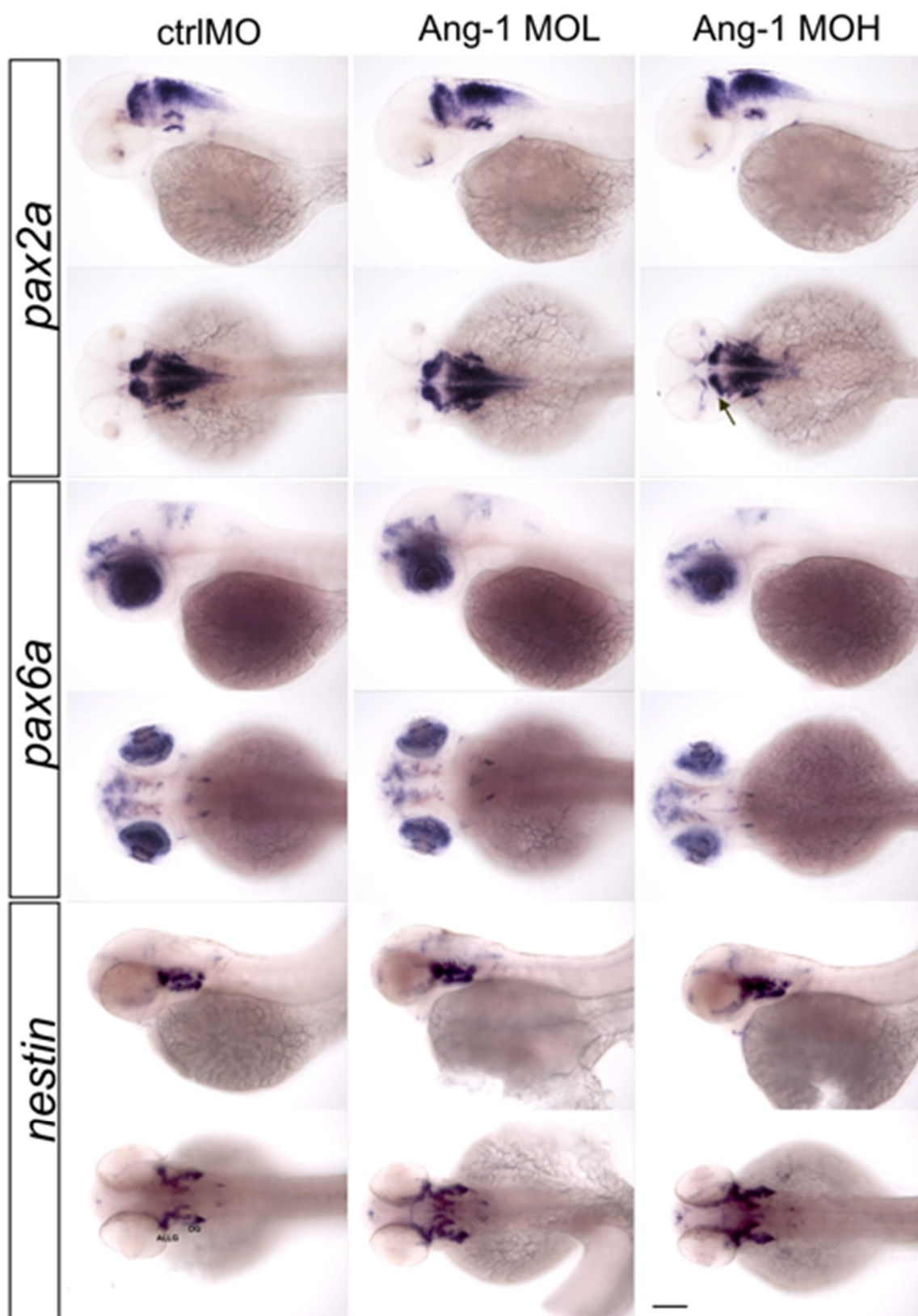

ALLG: anterior lateral line ganglion  
OG: octaval ganglion

Supplement: suppl Figure 2 [file rspb20150872supp3.pdf]
